# Patterns of Dyslipidemia Among Acute Coronary Syndrome (ACS) Patients at a Tertiary Care Hospital in Lahore, Pakistan

**DOI:** 10.7759/cureus.32378

**Published:** 2022-12-10

**Authors:** Muhammad Muneeb, Ammar H Khan, Attaullah Khan Niazi, Muhammad Usman Khan, Zanib J Chatha, Tahseen Kazmi, Noor Shahid

**Affiliations:** 1 Cardiology, Shalamar Medical and Dental College, Lahore, PAK; 2 Cardiology, Imran Idrees Hospital, Sialkot, PAK; 3 Cardiac Surgery, Shalamar Hospital Lahore, Lahore, PAK; 4 Cardiology, Shalamar Institute of Health Sciences, Lahore, PAK; 5 Community Medicine, Central Park Medical College, Lahore, PAK

**Keywords:** high-density lipoprotein cholesterol, triglyceride, acute coronary syndrome, myocardial infarction, unstable angina, dyslipidemia

## Abstract

Introduction: Dyslipidemia refers to the presence of abnormalities in lipid parameters. It has become a global issue with a high risk of cardiovascular diseases (CVDs). The aim of the investigation was to find out the pattern and prevalence of dyslipidemia among patients with the acute coronary syndrome (ACS).

Methodology: A cross-sectional study design was used. Data were collected using convenient sampling from 101 patients presenting with ACS, admitted at the critical care unit (CCU) / Rasheeda Begum Cardiac Centre (RBCC) of Shalamar Hospital, during a 12-month period from January 2020 to December 2021. Dyslipidemia is diagnosed by testing the lipid profile when there are one or more abnormal readings of the lipid profile.

Results: Nearly 43 (42.6%) had ST-segment elevation myocardial infarction (STEMI), 27 (26.7%) had non-ST segment elevation myocardial infarction (NSTEMI), and 31 (30.7%) were categorized as unstable angina (USA). Overall dyslipidemia was present in 84 (83.2%) patients. The prevalence of dyslipidemia was 55 (65%) in male patients and 29 (34.5%) in female patients. Dyslipidemia was present in 39 (90.7%) patients with STEMI, 25 (80.6%) in the USA, and 20 (74.1%) with NSTEMI.

Conclusion: The prevalence of dyslipidemia was quite high among ACS patients. The proportion of obese patients was also high in our study. However, dyslipidemia was more frequent in overweight patients.

## Introduction

Acute coronary syndrome (ACS) is defined as a range of clinical conditions characterized by a sudden reduction of blood flow to the heart. This may lead to permanent death of heart tissue and myocardial infarction. The term ACS is used in patients in which there is suspicion or confirmation of ischemia of heart muscles or infarction. This can be classified into non-ST-elevation myocardial infarction (NSTEMI), ST-elevation MI (STEMI), and unstable angina [1]. 

Dyslipidemia is an abnormal amount of lipids (e.g. triglyceride, cholesterol, and/or fat phospholipids) in the blood. It has become a global issue with a high risk of cardiovascular diseases (CVDs) [2]. The association of dyslipidemia with various CVDs has been observed in various studies around the world [2-3]. Due to the change in lifestyle factors like physical inactivity and dietary habits, it has become a public health concern [4]. Correction of dyslipidemia may lead to decreased CVD burden [2, 5]. Various randomized control trials and epidemiological studies suggested that a high level of low-density lipoprotein (LDL) was a prime risk factor and should be targeted to reduce cardiovascular disease [6-7]. LDL has been considered of prime importance for screening patients with dyslipidemia and coronary artery disease (CAD) [6]. High LDL concentrations are positively related to CAD development, and low HDL concentrations have been observed as one of the major risk factors for coronary atherosclerotic disease [8]. Dyslipidemia was identified as one of the most important modifiable risk factors for CAD [2, 6].

Increased total cholesterol (TC), LDL cholesterol, and non-elevated high-density lipoprotein (HDL) cholesterol were seen to correlate directly with mortality. In the Asian-Pacific region, one main reason was coronary heart disease (CHD). The major purpose of strategies to avoid CVDs is to reduce LDL with lipid-lowering therapies (LLTs). This might be due to the scarcity of strong evidence [7]. Reduced LDL levels may lead to a significant reduction in major cardiac events. The European Society of Cardiology (ESC) and the European Atherosclerosis Society (EAS) has recommended reducing LDL cholesterol levels to less than 70 mg/dL as the target for controlling dyslipidemia in patients with a high risk of cardiovascular disease [9]. 

Gaziano et al. showed for the first time that the triglyceride (TG)/HDL ratio was a solid forecaster of myocardial infarction. Furthermore, TG/HDL ratio is closely associated with cardiovascular risk factors and may predict CAD development and cardiovascular mortality. TG/HDL ratio also reflects the balance between atherogenic and protective lipoproteins [10]. Typical plaque rupture is rare in young patients with ACS; therefore, other risk factors apart from the conventional risk factors should be taken into account in these patients. However, in younger patients, we can consider the association of different parameters of metabolic syndrome (MS), i.e. larger waistline, hypertension (HTN), diabetes mellitus (DM), raised TG, and reduced levels of HDL. But in our work, we focused on the ratio of TG/HDL.

Dyslipidemia is a modifiable risk factor for most common CVDs; TG/HDL is unidentified until spotted during the initial presentation with ACS [11]. This study is about patterns and prevalence of dyslipidemia among ACS patients. Pakistan is facing a high burden of non-communicable diseases. This study will help in evidence-based medicine. Lipid profile has been recognized as the main culprit of increased cardiovascular events but observations in the local settings are rare. This study will fill the lacunae. The aim of this investigation was to find out the spectrum and frequency of dyslipidemia among patients with the ACS.

## Materials and methods

A cross-sectional study design was used. Data were collected using convenient sampling from 101 patients presenting with ACS admitted to the critical care unit (CCU) / Rasheeda Begum Cardiac Centre (RBCC), Shalamar Hospital, Lahore during a 12-month period from January 2020 to December 2021. History, clinical examination, and electrocardiography (ECG) were done and serum troponin was measured with fasting lipid profile during index admission or during the previous 30 days prior to admission. The exclusion criteria were stable ischemic heart disease (IHD), valvular heart disease, and cardiogenic shock/left ventricular failure (LVF). All patients with rales on lung auscultation decreased breath sounds on lung auscultation, Q waves, LV hypertrophy (LVH), and a widened QRS complex in ECG was classified as LVF in this study. The minimum sample size was calculated as 92 by using a 95% confidence coefficient, and 39% as the prevalence of dyslipidemia in IHD patients [12], adding 10% as the non-response rate, so it became 101. The data were collected using a self-designed questionnaire. Information about age, gender, lipid profile, and whether a coronary angiogram had been performed or not was taken. The Cronbach alpha showed sufficient reliability.

The study protocol was approved by the Shalamar Medical and Dental College Institutional Review Board (SMDC-IRB/AI/67/2020). Consent was taken from the patients before the collection of data. Dyslipidemia was diagnosed by testing the lipid parameters having one or more of the following disorders: blood cholesterol > 5.2 mmol/L (200 mg/dL), triglyceride > 1.7 mmol/L (150 mg/dL), LDL cholesterol > 2.58 mmol/L (100 mg/dL), very low-density lipoprotein (VLDL) normal range from 2 to 30 mg/dL, and HDL cholesterol < 1.03 mmol/L (40 mg/dL). The frequency and percentage of categorical variables were reported. The mean and standard deviation of the numeric variable were calculated. Data analysis was done using IBM SPSS 26.0 (IBM Corp., Armonk, NY).

## Results

The mean age of the patients who presented with ACS to the Medical ICU was 56.72 + 11.85 SD (in years). Out of the total patients, 68 (67.3%) were males and the remaining 33 (32.7%) were females. About 43 (42.6%) had ST-segment elevation myocardial infarction (STEMI), 27 (26.7%) have non-ST elevation myocardial infarction (NSTEMI), and 31 (30.7%) were categorized as unstable angina (USA). A coronary angiogram of 77 (76.2%) patients was done. The mean + SD of abdominal girth was 102.48 + 17.68 SD (cm). The mean + SD (units) of the lipid profile of the patients is given in Table 1.

**Table 1:** Lipid profile pattern of the acute coronary syndrome patients (n=101). TC, total cholesterol; TG, triglyceride; LDL, low-density lipoprotein; HDL, high-density lipoprotein; VLDL, very-low density lipoprotein *p-value<0.05 is statistically significant

Lipid Profile	Total	Male	Female	p-value
Total Cholesterol (TC)	157.50 + 49.86	154.75 + 48.03	163.15 + 53.77	0.43
Triglyceride (TG)	175.25 + 83.01	167.78 + 80.80	190.64 + 86.62	0.21
Low-Density lipoprotein Cholesterol (LDL)	104.12 + 40.08	103.75 + 43.26	104.88 + 33.17	0.90
High-Density lipoprotein Cholesterol (HDL)	35.79 + 10.78	35.07 + 9.57	37.27 + 12.95	0.34
Very-low Density lipoprotein (VLDL)	38.16 + 16.83	37.85 + 16.54	38.81 + 17.15	0.16

Overall dyslipidemia was present in 84 (83.2%) patients. The prevalence of dyslipidemia was seen in 55 (65%) male patients and 29 (34.5%) female patients. The distribution of dyslipidemia according to the lipid profile is given in Table 2. TG was >=150 mg/dL among 54 (53.5%) patients followed by LDL >=100 mg/dL among 51 (50.5%) patients. The mean TG/HDL ratio was 6.55 among ACS patients. The lower, middle, and upper quartiles of TG/HDL ratio were 2.97, 4.40, and 7.55.

**Table 2:** Patterns of dyslipidemia according to TC, TG, LDL, HDL, and VLDL (n=101). *p value less than 0.05 is statistically significant TC, total cholesterol; TG, triglyceride; LDL, low density lipoprotein; HDL, high density lipoprotein; VLDL, very low density lipoprotein

Lipid profile	Category (mg/dL)	n (%)	TG/HDL ratio	p-value
TC	< 200	85 (84.2%)	5.66	0.01*
	>= 200	16 (15.8%)	11.30	
TG	< 150	47 (46.5%)	2.89	<0.001*
	>= 150	54 (53.5%)	9.73	
LDL cholesterol	<100	50 (49.5%)	8.11	0.07
	>=100	51 (50.5%)	5.03	
HDL cholesterol	< 40	73 (72.3%)	8.06	<0.001*
	>=40	28 (27.7%)	2.62	
VLDL	2-30	50 (49.5%)	7.63	0.04*
	<2 or >30	51 (50.5%)	4.48	

In order to compare the TG/HDL ratio among patients with normal and abnormal lipid profiles, an independent sample t-test was used. TG/HDL was found statistically significant for patients with normal and abnormal TC, TG, HDL, and VLDL. TG/HDL ratio was significantly high among patients with TC greater or equal to 200 mg/dL. However, the difference between TG/HDL ratio among patients with less than 100 mg/dL and greater or equal to 100 mg/dL was statistically insignificant. TG/HDL ratio was higher among patients with normal VLDL. 

We observed that dyslipidemia was common among 72.3% of the patients with low HDL, 53.5% of the patients with high TC, and 50.5% of the patients with high LDL. Dyslipidemia was present in 39 (90.7%) patients with STEMI, 25 (80.6%) in the USA, and 20 (74.1%) with NSTEMI. Body mass index (BMI) less than 18.5 was taken as under-weight, 19-24.99 was normal, 25-29.99 was over-weight and greater and equal to 30 was used as obese. About 39 (38.6%) of the patients were obese, 35 (34.7%) were overweight, 26 (25.7%) were normal, and 01 (1.0%) were underweight. Out of the total patients who were obese, dyslipidemia was present in 29 (34.5%). Dyslipidemia was more frequent in overweight patients. Dyslipidemia was present in about 30 (35.7%) of the overweight participants, and 24 (28.6%) of the normal participants.

## Discussion

The current study investigated the patterns of dyslipidemia among patients presenting with ACS. We observed that dyslipidemia was present in 83.2% of cases in our study which was quite similar to another study conducted on diabetic patients in Pakistan. A recent study conducted in Pakistan among type 2 diabetic patients observed that 83.5% of the patients had dyslipidemia [13]. A recent study in Nepal observed an 88.1% prevalence of dyslipidemia [14]. A study reported that the prevalence of dyslipidemia was 48.6% which was quite low compared to our findings [11]. In Nepal, the prevalence of overall dyslipidemia was found to be 45.5% [15].

Another study conducted in Pakistan showed the prevalence of dyslipidemia among stroke patients. It showed the prevalence of dyslipidemia as 39.4% which was relatively low compared to our study [12]. A study investigated that 55.0% of the study population presenting with ischemic stroke showed lipid abnormalities [16]. Another study revealed the prevalence of dyslipidemia as 63.0% in the Pakistani population [17]. The findings of our study about the prevalence of dyslipidemia among patients with heart disease were quite high. Dyslipidemia was considered a modifiable factor for early diagnosis of abnormal heart functioning and for reducing the risk of stroke [16]. 

The prevalence of dyslipidemia in our study was 65.5% among male patients and 34.5% among female patients is almost double in male patients compared to female patients. The prevalence of dyslipidemia was 68.4% among men as compared to 43.7% in females in another study from Pakistan [12]. Dyslipidemia was found to be more common among male patients whereas the results of another study were dissimilar from our study that showed a statistically insignificant difference between male and female patients for dyslipidemia. The prevalence was 45.7% among male patients and 58.3% among female patients [11].

The prevalence of STEMI was higher in patients with dyslipidemia followed by unstable angina and NSTEMI. Among those patients who presented with STEMI, 39 (90.7%) were found to have dyslipidemia, 25 (80.6%) had dyslipidemia with unstable angina, and 20 (74.1%) had dyslipidemia with` NSTEMI. We observed that dyslipidemia was more common among patients with STEMI followed by unstable angina. The least common ACS was NSTEMI with dyslipidemia. A recent study done to observe the patterns and prevalence of dyslipidemia among ACS patients showed that dyslipidemia was highly prevalent among patients with unstable angina at 64.3% followed by NSTEMI at 48.1% and STEMI at 45.3% [11]. The findings for the pattern of dyslipidemia among patients with various instances of ACS in the study were opposite to our results.

In our study, the lipid abnormalities were a low level of HDL and a higher level of TC and LDL. A recent study reported that among the patients with dyslipidemia, 70.0% of the patients had high LDL, and 34.0% of patients with non-elevated HDL [12]. Another study observed that the most common abnormality was a low HDL ratio of 60.8% [11]. Another study conducted on the South Asian population stated that a low level of HDL was the strongest abnormality compared to others for dyslipidemia [18-19]. It was seen that the second most common lipid abnormality was a higher level of TC. A study conducted in Southwest Ethiopia reported that the most prevalent lipid abnormality was low HDL with 67.2% and high TC with 44.6% prevalence as the second most prevalent parameter [20]. Another study reported that a high level of TC was the third most common abnormality [21].

Some of the limitations of our study were cross-sectional/descriptive studies conducted in a single center with convenient sampling. Hence drawing a convenience sample and that too from the already diseased people presenting in the hospital may have its limitations. Moreover, there is no time dimension involved in cross-sectional studies and therefore no time interval between “exposure” and “outcome” [22].

## Conclusions

The prevalence of dyslipidemia was quite high among ACS patients. It was relatively more prevalent in patients with STEMI and overweight patients. Preventive strategies are required for obese, overweight, and STEMI patients. Dyslipidemia is an important risk factor in heart attacks and early detection can reduce the burden of morbidity, especially among high-risk cases. Moreover, associated risk factors such as obesity, overweight, and other general lipid abnormalities in ACS patients should be considered for the prevention of a rising trend of CVD and stroke among the Pakistani population.
